# Contagion of Temporal Discounting Value Preferences in Neurotypical and Autistic Adults

**DOI:** 10.1007/s10803-021-04962-5

**Published:** 2021-04-02

**Authors:** Louisa Thomas, Patricia L. Lockwood, Mona M. Garvert, Joshua H. Balsters

**Affiliations:** 1 Department of Psychology, Royal Holloway, University of London, Egham, TW20 0EX Surrey UK; 2 Department of Experimental Psychology, University of Oxford, Oxford, UK; 3 Department of Psychology, Max Planck Institute for Human Cognitive and Brain Sciences, Leipzig, Germany; 4 Wellcome Centre for Integrative Neuroimaging, Department of Clinical Neurosciences, FMRIB, University of Oxford, Oxford, Nuffield UK; 5 Present: Centre for Human Brain Health, School of Psychology, University of Birmingham, Birmingham, UK

## Abstract

Neuroeconomics paradigms have demonstrated that learning about another’s beliefs can make you more like them (i.e., contagion). Due to social deficits in autism, it is possible that autistic individuals will be immune to contagion. We fit Bayesian computational models to a temporal discounting task, where participants made decisions for themselves before and after learning the distinct preferences of two others. Two independent neurotypical samples (N = 48; N = 98) both showed a significant contagion effect; however the strength of contagion was unrelated to autistic traits. Equivalence tests showed autistic (N = 12) and matched neurotypical N = 12) samples had similar levels of contagion and accuracy when learning about others. Despite social impairments being at the core of autistic symptomatology, contagion of value preferences appears to be intact.

**Supplementary Information:**

The online version contains supplementary material available at 10.1007/s10803-021-04962-5.

Social influence has been shown to bias our behaviours and preferences (Behrens et al., 2008; Cialdini & Goldstein, 2004; Izuma, 2013; Meyer et al., 2019; Raafat et al., 2009; Shamay-Tsoory et al., 2019). For example, multiple studies have demonstrated that learning the alternative value preferences of another person significantly shifts our own value preferences – a phenomenon referred to as contagion (Apps & Ramnani, 2017; Garvert et al., 2015; Nicolle et al., 2012; Reiter et al., 2019; Suzuki et al., 2016; Wheeler, 1966). Given that autistic individuals often struggle to understand the alternative preferences of others (Baron-Cohen et al., 1985; Hamilton, 2009), it is possible that they are immune to the effects of contagion or may experience contagion to a lesser degree than neurotypical (NT) individuals. Here, we use Bayesian modelling to investigate contagion of value preferences in autistic individuals and autistic traits in the NT population.

One computational framework increasingly employed in cognitive and social neuroscience is Bayesian belief updating (Bennett, 2015). Previous research has found that Bayesian associative processes underlie learning about environmental features as well as more complex social values (Behrens et al., 2008; Bennett, 2015; Campbell-Meiklejohn et al., 2010; Mussey et al. 2015; Perreault et al., 2012; Suzuki et al., 2012). Within a Bayesian framework, contagion can be interpreted as an integration of an individual’s prior beliefs and their beliefs about newly presented social information (i.e., from another individual or group) to form a new posterior belief. Measuring social influence using a Bayesian framework not only helps us to define our concepts more clearly, but it also allows us to precisely quantify observed behaviours. Statistical model comparisons suggest that Bayesian approaches outperform other computational models of social influence, by additionally incorporating information about the credibility of external sources of information (De Martino et al., 2017; Park et al., 2017), as well as the participant’s degree of uncertainty in their initial beliefs (Moutoussis et al., 2016), using the precision (i.e., the width) of priors.

Bayesian frameworks (also called predictive coding accounts) have also been employed to explain behaviours in autism spectrum conditions (ASC). Predictive coding accounts of ASC suggest that autistic individuals struggle to generate precise internal prior beliefs about the world (both social and non-social; Karvelis et al., 2018; Lawson et al., 2017; Pellicano & Burr, 2012; Van De Cruys et al., 2014). Pellicano et al. (2011) found that autistic children took longer to learn reward probabilities than typically developing children in a foraging task and were less consistent and optimal in their search strategy, indicating a deficit in rule learning. A more recent study by Lawson et al. (2017) found differences in learning of volatility, with autistic adults being more likely than NT adults to overlearn about environmental volatility. This resulted in less surprise when outcomes were unexpected, relative to expected. A decrease in surprise at unexpected outcomes was also related to greater symptom severity. Similar findings have been uncovered in NT individuals with higher levels of autistic traits. Karvelis et al. (2018) explored predictive coding in NT individuals using a visual learning task and showed that increased autistic traits in this sample corresponded to less precise prior expectations, combined with more precise sensory representations. Taken together, these findings suggest a deficit in generating internal priors that form the prediction of reward outcomes in autistic individuals relative to NT controls, and that this finding can also be observed in NT individuals with higher levels of autistic traits. This may translate to decreased learning about the subjective values of others in tasks measuring contagion.

Several studies have shown contagion of subjective value preference within NT samples, using temporal discounting (TD) tasks. In these studies, participants made TD choices on behalf of themselves and others with alternative value preferences. After learning the alternative value preferences of another individual, or group, the participant’s own discount rate changed to become more like that of the other (Apps & Ramnani, 2017; Garvert et al., 2015; Nicolle et al., 2012). Research into discounting in ASC is mixed. Some studies suggest that TD follows the same pattern as in NT individuals (Antrop et al., 2006; Demurie et al., 2012; Warnell et al., 2019), whereas others have found that autistic individuals discount future rewards more steeply than NT controls (i.e., are more impatient; Carlisi et al., 2017; Chantiluke et al., 2014). To account for individual differences in contagion, the value preferences of the other agents in this study vary in line with the participant’s own preferences.

Garvert et al. (2015) suggest that differences between self and other value preferences (reflected in a prediction error signal in ventral striatum) modulate the individual’s own internal value representation (i.e., plasticity within the vmPFC) in the TD contagion task. The extent to which plastic changes occurred within the vmPFC corresponded to the strength of the contagion effect. This is particularly relevant in light of the results of Balsters et al. (2017), who found that autistic individuals do not produce prediction error signals about others (i.e., a brain signal describing the difference between expected and unexpected outcomes for another person). The absence of this social prediction error signal could make autistic individuals immune to contagion effects, as appropriate social prediction error signals should be necessary for learning about another person. In addition, autistic adults have been shown to be more consistent and inflexible in their choices than NT controls in a probability discounting (PD) task (Wu et al., 2018). A preference for predictability and sameness has also been found in NT individuals with higher levels of autistic traits (Goris et al., 2019). In this study, significantly positive correlations were found between the level of autistic traits and preference for both more predictable music pieces, and for visual items that were increasingly similar to a visual prime. Participants with higher levels of autistic traits were also faster to choose decks of cards with predictable outcomes, indicating an implicit preference for the more predictable decks. Autistic participants have also been found to revert to previously preferred responses more quickly than NT controls in a probability reversal learning task with feedback provided on an 80:20 schedule (D’Cruz et al., 2013). Whilst there were no differences in learning of reward contingencies in this study, autistic participants were also faster than NT controls to revert to the previously rewarded response following incorrect feedback. Together, these studies indicate that the internal value preferences of autistic individuals, and NT individuals with higher levels of autistic traits, may not change due to the influence of the other, or that these participants may revert to their own preferences more quickly after making choices on behalf of another individual.

We conducted three studies to examine: (1) whether NT individuals shift their preferences when they learn about other people’s preferences, (2) whether levels of autistic traits in the NT population are associated with contagion of value preferences, and (3) whether autistic adults show comparable shifts in preference to NT controls. In Study 1, participants completed a TD contagion task as well as a measure of autistic traits. In Study 2, we tested a larger replication sample of NT participants at a separate research site. Finally, in Study 3, we tested a sample of autistic adults on the TD contagion task. In all three studies, we conducted separate analyses on contagion for more impulsive and more patient agents, as previous research has suggested contagion effects are stronger when presented with a more patient agent (Moutoussis et al., 2016) using a similar paradigm.

We predicted that we would observe contagion effects in both the NT samples, such that people would shift their preferences after learning about another person’s preferences. We also predicted that levels of autistic traits in the NT samples would be associated with lower levels of contagion, and that autistic adults would be less accurate at learning the value preferences of the other agents. This reduced learning would result in less contagion of value preferences relative to NT adults.

## Methods

### Participants

#### Study 1

A sample of 49 NT participants (23 male, 24 female, 2 unreported, mean age = 23.73, SD $\pm$ 3.86) were recruited from the University of Oxford. Participants were paid at a rate of £10 per hour and were told that they would receive an additional bonus based on a randomly selected trial from the experiment. In fact, participants were paid a randomly selected bonus ranging between £1 and £10 on the day and were informed that a trial had been chosen. Informed consent was obtained, and ethical approval was granted by the Departmental Ethics Committee at Oxford.

#### Study 2

One hundred NT participants (60 female, mean age = 21.35, SD $\pm$ 2.11) volunteered to take part, and were recruited from the Royal Holloway, University of London campus. As an incentive for participation, participants were invited to enter into a prize draw to win an Amazon voucher for the amount of one of their chosen monetary outcomes (£1–20), which was drawn when data collection commenced. Immediate amounts (e.g., £3 now) were sent immediately, and delayed amounts (e.g., £8 in two weeks) were sent following the specified delay, with a notification of the win sent immediately following the draw. Informed consent was obtained, and Ethical approval was granted by the Royal Holloway Departmental Ethics Committee.

#### Study 3

A further sample of 14 participants with a diagnosis of an ASC (8 female, mean age = 22.29, SD $\pm$ 3.36) were recruited from the Royal Holloway, University of London campus, and externally. Although this sample is smaller than the Study 1 and Study 2 samples, a power analysis indicated that a sample of four was required to achieve 80% power when examining an effect of contagion in previous discounting tasks. Therefore, the sample of 14 included here maximises power and allows for exclusions and potential dropouts. Participants in this sample were also invited to enter into the same prize draw as the participants in Study 2 to win an Amazon voucher for the amount of one of their chosen monetary outcomes (£1–20). An additional three participants were recruited to this study following the prize draw and were paid £8 for their time. Informed consent was obtained, and Ethical approval was granted by the Royal Holloway Departmental Ethics Committee.

Scores on the autism quotient (AQ; Baron-Cohen et al., 2001), scored using the Likert scoring method (Austin, 2005) are presented in Table 1 for the final samples for all three study groups, and the Study 1 and Study 2 samples combined (following the exclusions outlined at the end of the Methods section). See Supplemental Table 1 for AQ data scored using the binary scoring method (Baron-Cohen et al., 2001) for all three samples.

**Table 1:** Descriptive statistics (mean (SD ±)) for AQ subscale scores and total scores for both NT samples (Study 1 and Study 2), and the ASC sample (Study 3), following exclusions

	Study 1	Study 2	Study 1/Study 2 combined	Study 3
AQ Social Skills	25.87 (3.21)	20.56 (3.99)	22.28 (4.50)	29.83 (4.13)
AQ Attention Switching	26.94 (3.30)	24.46 (3.97)	25.26 (3.93)	33.17 (3.66)
AQ Attention to Detail	25.61 (3.90)	24.85 (4.36)	25.10 (4.21)	29.42 (4.58)
AQ Communication	24.89 (4.54)	19.82 (3.79)	21.47 (4.68)	31.67 (3.89)
AQ Imagination	25.78 (3.82)	19.81 (4.04)	21.75 (4.85)	25.42 (3.83)
AQ TOTAL	129.09 (10.41)	109.51 (13.13)	115.85 (15.34)	149.50 (14.39)

### Discounting Tasks

#### Study 1

Participants first completed a TD contagion task. This was presented in MATLAB and was developed using the experimental script programmed by Mona Garvert (Garvert et al., 2015), using the Cogent 2000 v125 graphics toolbox. Participants made a series of self-paced hypothetical choices between two monetary values (£1–20) presented simultaneously, choosing between a small amount of money available immediately (e.g., £3 now), or a larger amount available after a specified delay period (tomorrow, 1 week, 2 weeks, 4 weeks, 6 weeks, 2 months, or 3 months). Responses were made using left and right arrow keys, corresponding to the location of the choice on the screen. The side (left or right) of the immediate option was randomised on a trial-by-trial basis.

**Fig. 1:**
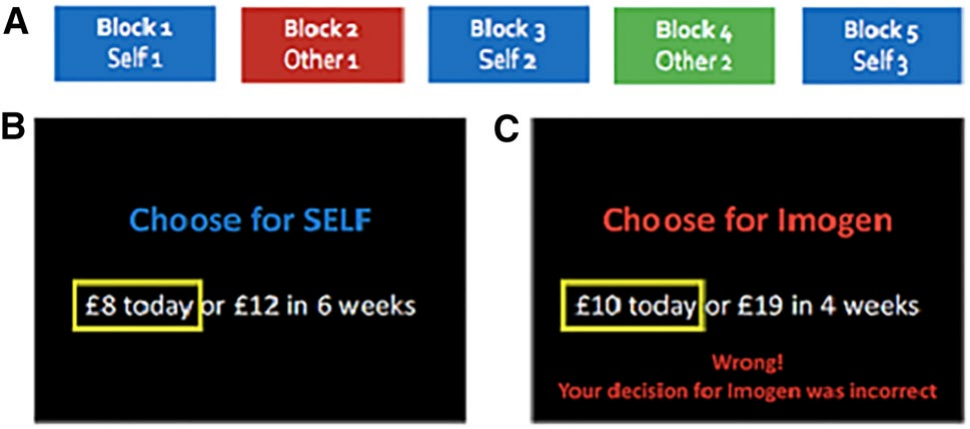
Experimental design. **a** Shows the order and type of the five blocks for each task. The Self, Other1 and Other2 colours were randomised across participants (RGB colours). **b** Shows an example experimental screen for Self-trials, participants saw the type of trial (Self or Other), indicated by the name in colour, along with the options. The yellow box appeared around the participant’s selected choice, following key press. **c** Shows an example experimental screen for Other-trials, which follow the same format as Self-trials, with additional feedback. This feedback was presented on screen 500 ms after the yellow box appeared, and was displayed for 1000 ms before the screen cleared for the next trial

The task was divided into five blocks of 50 trials (Self1, Other1, Self2, Other2, and Self3), with a self-timed break after 25 trials. During Self-trials, participants were instructed to choose for themselves according to their own preferences, as they believed that one of these choices would be selected as their potential bonus payment. In blocks two and four (i.e., Other1-block and Other2-block), participants made choices on behalf of two simulated agents, and were informed that these were the choices of two previous participants. In fact, the behaviour of these two agents was modelled online based on the participants own choices in the first block (i.e., Self1), to be plus or minus one of the participants own discount rate. The direction (± 1) of Other1 and Other2 was counterbalanced across participants within the script (see the Estimation of discount rates and simulation of the Other’s choices section below for details of this calculation). Two gender-matched names (or two randomly selected names for participants who did not report their gender) were chosen to represent these two agents. During all Other-trials, feedback was also displayed on a trial-by-trial basis, to inform participants whether their choice for the other agent was correct. If participants chose the option that would be preferred by this modelled agent, feedback was displayed stating that the choice was correct, or incorrect if the participant chose the other option. This feedback remained on the screen for 1000 ms. Task order and example experimental screens are shown in Fig. 1. See Supplemental Information for details of how choice pairs were generated for all blocks.

#### Study 2 and 3

Participants completed a TD contagion task which followed the same format as the Study 1 task, and the Garvert et al. (2015) task, with five trial blocks (Self1, Other1, Self2, Other2, and Self3), and two simulated agents. The number of trials in each block was reduced in this task from 50 to 30, and participants received a self-timed break after 15 trials. Participants also completed a PD contagion task as part of a separate project. The order of presentation (i.e., TD contagion task then PD contagion task or vice versa) was counterbalanced across participants.

Participants in Study 2 and 3 were once again informed that the choices of the two other agents were the choices of two previous participants. In fact, the names of these two agents were chosen at random from a list of popular UK names (50 of each gender, with a different random allocation for each task), and were gender-matched to the participant. The choices of these two agents were modelled following the same procedure as Study 1.

### Estimation of Discount Rates and Simulation of the Other’s Choices

#### Study 1, 2, and 3

A log hyperbolic model was fitted to participant’s choices, and the choices of the two modelled agents were also produced using this model. The subjective value of each choice was calculated on each trial according to the following equation:

1 $$V= \frac{M}{1+kD}$$

In this equation, subjective value, and the reward magnitude are represented by V, and M respectively, *k* represents the agent’s discount rate, and *D* indicates the delay period in days. The log *k* parameter values were set between -4 and 0. A log* k* value of -4 indicates that the individual is barely sensitive to delay and bases their decision only on reward magnitude. As log *k* approaches 0, individuals are more sensitive to delay and discount rewards more steeply as a function of time (Carlisi et al., 2017; Garvert et al., 2015). The value of the immediate option will henceforth be referred to as ${V}_{SS}$, and the value of the delayed option will be referred to as ${V}_{LL}$. The subjective value of ${V}_{SS}$ will always correspond to the magnitude of the reward (*M*), because the delay period in days (*D*) is 0.

The following softmax function transformed the subjective values of each option into choice probabilities (i.e., ${P}_{SS}$ and ${P}_{LL}$):

2 $${P}_{LL}= \frac{1}{{1+e}^{-\beta ({V}_{LL}- {V}_{SS})}}$$

In this equation, β is a free parameter, which characterises noise in an individual’s choices. Log β parameters were set between -1 and 1. Values closer to -1 indicate larger non-systematic deviations around the indifference point (i.e., the point at which both choice options are equally preferred).

During Other-trials, choices were modelled to reflect the preferences of an agent whose discount rate (log *k*) was plus one or minus one from the participant´s own log *k* (calculated within the experimental script, based on the Self1-block). Differences in subjective value of the two options were translated into choice probabilities using the softmax function (Eq. (2)) with the temperature parameter β fixed at 1. The direction (± 1) of Other1 and Other2 was counterbalanced across participants within the experimental script. The agent with a larger discount rate discounts rewards more steeply than the participant and tends to prefer options that are immediately available (i.e., more impulsive), whereas the agent with the smaller discount rate waits longer for rewards than the participant (i.e., more patient).

Participants’ own log *k,* and log β values were also derived from Eqs. (1), and (2), and were updated on a trial-by-trial basis using a Bayesian model. A uniform prior was updated on each trial, and the posterior was calculated as the likelihood of an individual’s choice given the parameters log *k*, and log β weighted by this prior. This posterior was then set as the prior for the next trial and was updated on a trial-by-trial basis. To avoid the influence of strong priors for the other agents on the estimation of priors for Self, priors were reset at the beginning of the Self2 and Self3 blocks. As such, the posterior from the end of the Self1-block then became the prior for the start of the Other1-block, and the posterior from the end of the Self2-block then became the prior for the start of the Other2-block. This is in line with previous studies that have shown that we use our own priors as the starting point for learning about others (Lockwood et al., 2018; Tarantola et al., 2017). The log β parameter was set to start at 0.3 for Self-blocks and was fixed at 1 for all Other-blocks.

### Contagion When Accounting for Uncertainty

#### Study 1, 2, and 3

**Fig. 2:**
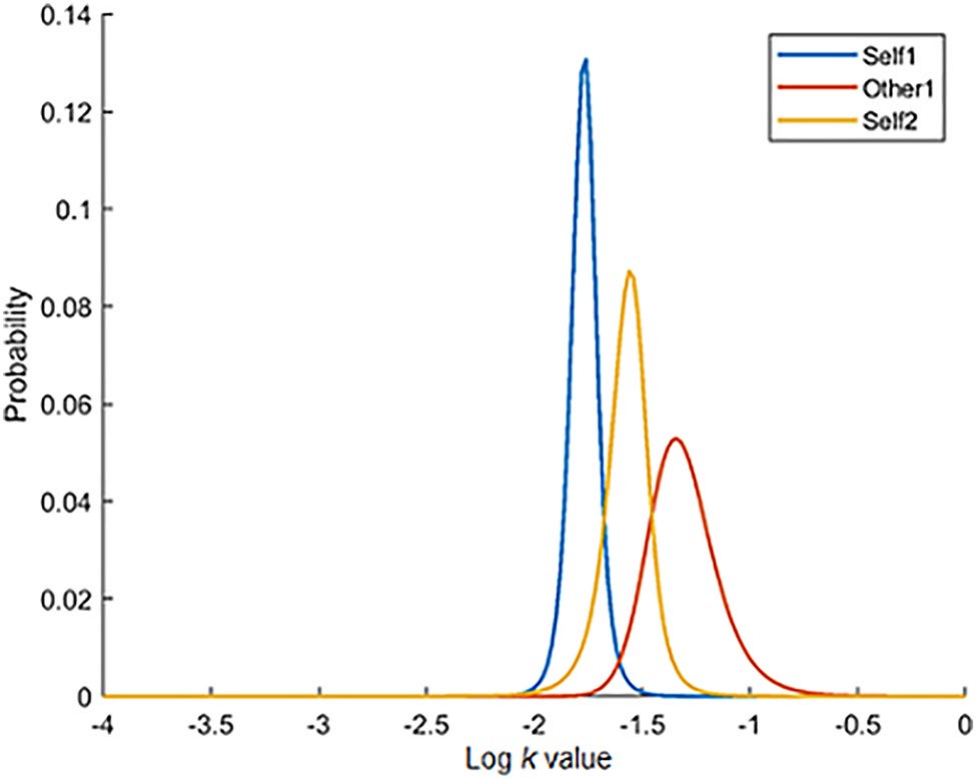
Example participant behaviour: These three Gaussian distribution curves show the posterior distribution at the end of the Self1-, Other1-, and Self2-blocks for an example participant (Study 2 sample). At Self2, the posterior has shifted towards the posterior for Other1 (i.e., contagion)

A Bayesian belief update measure was used to calculate the change in participants’ log *k* values after learning about the preferences of the two other agents (i.e., contagion). The change in priors was calculated using Kullback–Leibler divergence ($D_{KL}$), which quantifies the divergence in the distribution of two data sets (Kullback & Leibler, 1951), and accounts for both the change in the overall peak of this distribution, and the precision of the distribution. The precision reflects an individual’s confidence (or certainty) in their belief (Moutoussis et al., 2016). In the studies presented here, $D_{KL}$ quantifies the divergence in the posterior distribution between the end trials of two blocks, following trial-by-trial update of the prior. See Fig. 2 for these posterior distributions plotted for an example participant. $D_{KL}$ was normalised for each analysis, such that changes in log *k* in the same direction as the other agent (e.g., the participant’s log *k* became more positive (i.e., impulsive) after making choices on behalf of a more impulsive agent) resulted in positive $D_{KL}$ values, and changes in log *k* in the opposite direction resulted in negative $D_{KL}$ values. $D_{KL}$ was also sorted and analysed separately for impulsive and patient others (i.e., $D_{KL}$ for a more positive/impulsive or a more negative/patient agent). Shift variables (as per Garvert et al., 2015) are outlined and included in the Supplemental Information for comparison.

### Individual Differences in Social Cognition Self-Report Measures

#### Study 1, 2, and 3

Participants also completed a block of six individual differences questionnaire measures. In Study 1, these were filled out following completion of the TD task. In studies 2, and 3, these were filled out in between the two discounting tasks (TD and PD). The questionnaire block (which included the AQ; Baron-Cohen et al., 2001) was presented full screen using Qualtrics. Participants completed the questionnaires in the following order: Social Network Index (SNI; Cohen et al., 1997), Apathy Motivation Index (AMI; Ang et al., 2017), Questionnaire of Cognitive and Affective Empathy (QCAE; Reniers et al., 2011), Twenty-Item Toronto Alexithymia Scale (TAS-20; Bagby et al., 1994), Short Form Self-Report Psychopathy scale (SRP-SF; Gordts et al. 2017), and AQ.

AQ data are analysed in relation to contagion variables in this paper, and data from the remaining questionnaires are not included, as this data was collected for a collaboration project. A Likert scoring method (scored 1–4, as introduced by Austin, 2005) is used here for scoring the AQ, instead of the original binary scoring method (Baron-Cohen et al., 2001). Higher internal consistency and greater test–retest reliability has been found for the four-point Likert scoring method compared to binary scoring using a sample of NT participants (Stevenson & Hart, 2017). As in the original measure, higher scores indicate a greater number of autistic traits. In Study 1, and 2, individual differences are indexed using the subscale scores on the AQ. Likert-scored AQ scores for the final samples (following the exclusions outlined at the end of the Methods section) for all three study groups are presented in Table 1, and binary-scored AQ scores are presented in Supplemental Table 1.

### Awareness of Manipulation and Behavioural Change

#### Study 2, and 3

Two questions (with *“yes”* or *“no”* responses) were added to studies 2 and 3 to determine whether participants believed that the other agents were real participants (i.e., were unaware of the manipulation; *“Did you believe that the other two players were real participants?”*), and whether they were aware of their behaviour changing (*“Did you notice yourself changing your choices throughout the experiment?”*). These questions were asked following completion of both discounting tasks and the questionnaire block. If answering “*no*” to the first question, participants were prompted to provide a reason.

### Participant Exclusions

#### Study 1

One outlier was excluded from this sample (i.e., $\pm$ 3 SDs of $D_{KL}$). Four participants in the final sample with log *k* values close to -4 also had two other agents with behaviour (log *k*) in the same direction. For these participants, data were analysed for the other agent (Other1 or Other2) with the greatest distance between the participant’s own discount rate, and the model discount rate. Data from 48 participants were entered into the final analysis.

#### Study 2

Two outliers (using the same criteria as Study 1) were excluded from this sample. From the final sample, three participants had two agents with behaviour in the same direction. As in Study 1, data were analysed for the other agent with the greatest distance between the participant’s own discount rate, and the model discount rate. Data from 98 participants were entered into the final analysis.

#### Study 3

Two outliers were excluded, and data were analysed for only one agent for two participants in the final sample. Data from 12 participants were included in the final analysis.

### Statistical Analyses

Analyses for all three studies are run in log space. All analyses were run using Jamovi (version 1.1.9), JASP (version 0.13.0.0), and R (version 3.5.2) in RStudio (version 1.1.463). G*Power (version 3.1) was used for power calculations, and figures were produced in R. Bayes factors (calculated using Jamovi jsq package presets) are reported for all analyses and are interpreted in line with the jsq package criteria. Bayes factors allow the user to quantify the evidence in favour of one hypothesis over another (e.g., the hypothesis of interest versus the null), given the data (Kass & Raftery, 1995; Quintana & Williams, 2018). In the analyses presented below, ${BF}_{10}$ is used for significant analyses and denotes the likelihood that the hypothesis of interest is correct. ${BF}_{01}$ is used for non-significant analyses and denotes the likelihood that the null hypothesis is correct.

#### Study 1, and 2

To determine whether a significant contagion effect was present, separate one-sample t-tests (or Wilcoxon signed-rank tests for non-normally distributed data) were run on $$ D_{KL} $$ data (separately for more patient and more impulsive agents). Robust repeated-measures t-tests were then run on $$ D_{KL} $$ data to assess the effect of direction (i.e., patient versus impulsive other) on contagion. Repeated-measures t-tests were also used to assess the effect of direction on accuracy in these samples, and correlation analyses were performed to determine whether a relationship could be observed between accuracy and $$ D_{KL}.$$ Exploratory independent-measures t-tests were conducted to determine gender effects on TD task parameters (i.e., log *k* and log $$\upbeta$$). To assess the association between autistic traits (as measured by the AQ) and contagion in the two NT samples, data were collapsed across these samples, and entered into two regression analyses.

Further analyses (robust independent-measures t-tests) were conducted on the Study 2 data only, to determine whether awareness of manipulation and behavioural change (assessed by the final two questions included in this study) were related to $$ D_{KL}.$$

#### Study 3

A subset of NT participants from the Study 2 sample were selected for comparison with the Study 3 ASC sample, and the two groups were compared using Yuen’s robust independent-samples t-tests on the following variables: AQ total score, AQ subscales, age, log *k*, log β, $D_{KL}$, and accuracy. The two groups were also tested for equivalence across samples using Bayesian independent-measures t-tests for both accuracy and $D_{KL}$ variables.

## Results

Summary descriptive statistics for all following analyses are presented in Table 2, descriptive statistics for the AQ subscales and total scores for all three samples are included in Table 1. Bayes factors and interpretations are included in Supplemental Table 2. Analyses for the Study 1 and 2 NT samples are presented together, and analyses for the Study 3 ASC sample are presented separately.

**Table 2:** Descriptive statistics (mean (SD $\pm$)) for the final samples (following exclusions) for both NT study samples (Study 1 and Study 2) and the ASC study sample (Study 3)

	Study 1 (N = 48)	Study 2 (N = 98)	Study 3 (N = 12)
Age	23.81 (3.86)	21.41 (2.09)	22.33 (3.63)
Gender	23 female	59 female	8 female
Log *k*	− 2.17 (.84)	− 1.68 (.67)	− 2.28 (.78)
Log β	− .13 (.27)	− .19 (.35)	− .08 (.52)
$D_{KL}$– impulsive (log *k* + 1)	.96 (3.08)	.31 (2.39)	1.50 (3.31)
$D_{KL}$– patient (log *k* − 1)	2.14 (3.44)	.90 (2.45)	1.36 (3.57)
Percent Accuracy – impulsive (log *k* + 1)	80.18 (5.65)	74.46 (11.18)	75.33 (8.92)
Percent Accuracy – patient (log *k* − 1)	83.79 (6.05)	78.60 (10.30)	79.72 (12.75)

### Social Contagion When Accounting for Uncertainty (*D*_*KL*_)

**Fig. 3:**
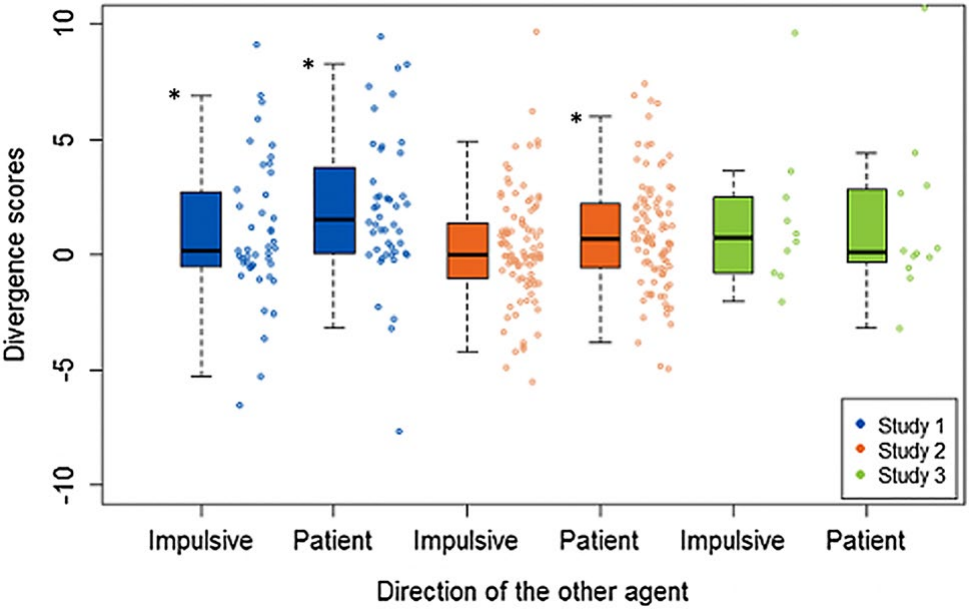
Group comparisons of $D_{KL}$ scores: Descriptive statistics for all divergence score ($D_{KL}$) variables for the two NT samples (Study 1 and Study 2), and the ASC sample (Study 3) are presented here, split for more impulsive (log *k* + 1) and more patient (log *k* -1) agents. Positive values indicate a shift towards the TD preferences of the other agent (i.e., contagion), whereas negative values indicate a shift away. One-sample t-tests (or Wilcoxon signed-rank tests where there were non-normal distributions) were conducted on Study 1 and Study 2 data, and significant results (*p* < 0.05) are indicated with an asterisk

To determine whether there was a significant change in behaviour after learning the value preferences of another person (i.e., contagion), one-sample t-tests were run on the Study 1 and Study 2 sample data. If data were not normally distributed (determined by Shapiro–Wilk tests of normality, see Supplemental Information), Wilcoxon signed-rank tests were used. Tests were conducted separately for more impulsive agents (log *k* + 1) and more patient agents (log *k* -1). See Table 2 and Fig. 3 for descriptive statistics (mean and SD) for the $D_{KL}$ variables for all three study samples. Contagion was significant after learning about patient others in both the Study 1 (W (47) = 1005, *p* < 0.001, $\text{d}$= 0.62, ${BF}_{10}$= 284.98) and Study 2 (t (94) = 3.60, *p* < 0.001, $\text{d}$= 0.37, ${BF}_{10}$= 41.76) samples. Contagion was also significant for impulsive others in the Study 1 sample (t (43) = 2.05, *p* = 0.046, $\text{d}$= 0.31, ${BF}_{10}$= 1.10), but not in the Study 2 sample (W (97) = 2707, *p* = 0.319, $\text{d}$= 0.13, ${BF}_{01}$= 4.10).

To determine whether there was an effect of direction (e.g., greater shifts in behaviour after learning about a more impulsive versus a more patient other) robust repeated measures t-tests were performed on the $D_{KL}$ values to allow for non-normally distributed data. There was a significant effect of direction in the Study 1 sample (t (27) = -2.07, *p* = 0.048, $\text{d}=$ 0.32, ${BF}_{10}$ = 0.91), with a stronger contagion effect for more patient others. There was no significant effect of direction on $D_{KL}$ values in the Study 2 sample (t (56) = -1.57, *p* = 0.121, $\text{d}=$ 0.19, ${BF}_{01}$ = 2.95).

These results demonstrate a significant contagion effect in both Studies 1 and 2, indicating that participants were influenced by the choices of the other agents. Although individuals did show greater contagion when exposed to a more patient other, this effect was only significantly greater than contagion to a more impulsive agent in the Study 1 sample, and the Bayes factor for this finding suggests that the effect is anecdotal.

### Accuracy When Making Choices for the Other Agent

Repeated measures t-tests were used to determine whether there was a significant effect of direction on accuracy. In the Study 1 sample, there was a significant effect of direction on accuracy (t (43) = -2.50, *p* = 0.016, $\text{d}$ = -0.38, ${BF}_{10}$ = 2.60), with greater accuracy when making choices on behalf of more patient agents (log *k* -1) versus more impulsive agents (log *k* + 1). This finding was replicated in the Study 2 sample (t (94) = -2.71, *p* = 0.008, $\text{d}$ = -0.28, ${BF}_{10}$ = 3.51). As in the Study 1 sample, accuracy was higher for patient agents versus impatient agents. See Table 2 for descriptive statistics including percentage accuracy, which accounts for the different number of trials across samples (50 trials per block in Study 1, and 30 trials per block in Studies 2 and 3).

**Fig. 4:**
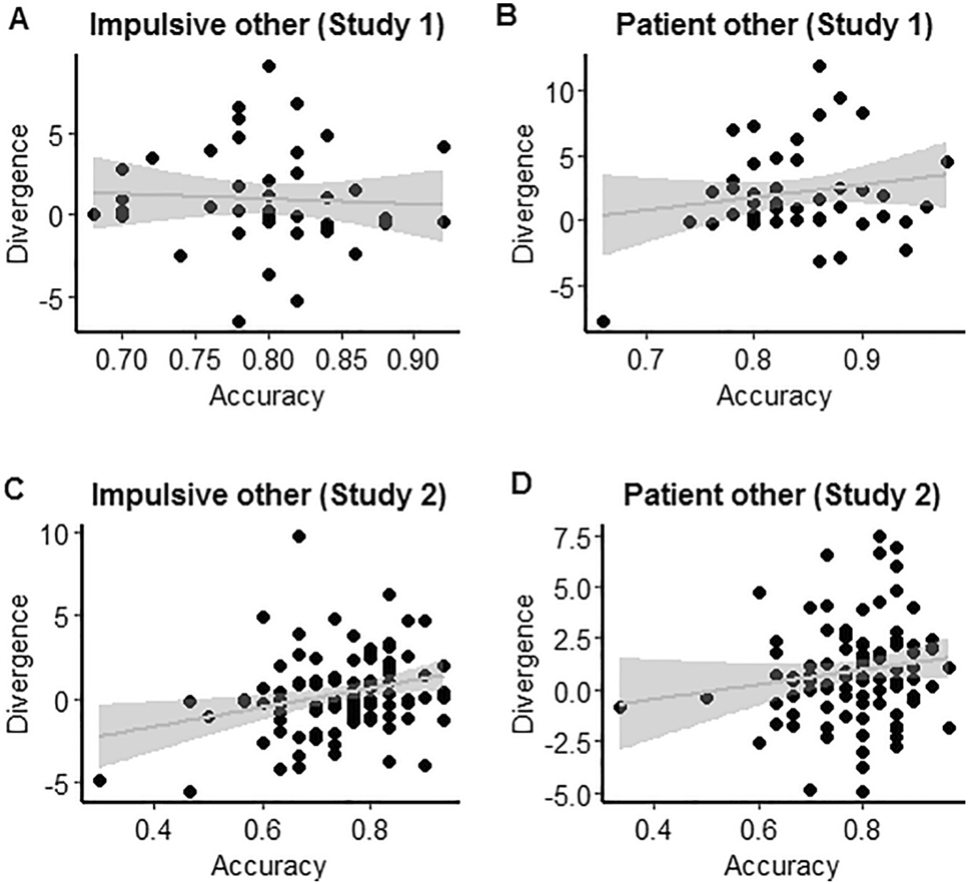
Correlations between percentage accuracy and $D_{KL}$. **a** Shows the correlation for more impulsive agents in the Study 1 sample, and **b** for more patient agents in the Study 1 sample. **c** Shows the correlation for impulsive agents in the Study 2 sample, only this correlation was significant. **d** Shows the correlation for patient agents in the Study 2 sample

To determine whether accuracy was related to the strength of contagion, correlation analyses between $D_{KL}$ and accuracy were run for each direction for the two NT samples. In the Study 1 sample, the correlation between $D_{KL}$ and accuracy was not significant for impulsive (*r* = -0.06, *p* = 0.708, ${BF}_{01}$ = 4.97), or patient other agents (*r* = 0.17, *p* = 0.243, ${BF}_{01}$ = 2.86). In the Study 2 sample, there was a significant positive correlation showing that stronger contagion ($D_{KL}$) was associated with greater accuracy for impulsive agents (*r* = 0.27, *p* = 0.007 ${BF}_{10}$ = 4.56; Fig. 4), although the correlation was not significant for more patient agents (*r* = 0.15, *p* = 0.149, ${BF}_{01}$ = 2.79).

Whilst these findings indicate that NT participants were more accurate at making choices on behalf of more patient agents, versus more impulsive agents, Bayesian support for these findings was anecdotal. As the correlation between contagion ($D_{KL}$) and accuracy was only significant for the more impulsive agent in the Study 2 sample (with moderate Bayesian support), these findings suggest that there is not a reliable relationship between accuracy and contagion in NT individuals.

### Awareness of Manipulation and Change, and Effect on Contagion

From the final sample (Study 2 participants only), a total of 87 participants completed the awareness of manipulation question (40.23% believed manipulation), and 74 participants completed the change awareness question (56.76% noticed their behaviour changing). To determine whether participant’s answers to these questions were related to contagion, two robust independent measures t-tests were run, on mean $D_{KL}$ variables.

Whilst the percentage of participants believing the manipulation is close to chance, there was no significant effect of awareness of manipulation on mean $D_{KL}$ (t (40.30) = 1.24, *p* = 0.223, $\xi$= 0.22, ${BF}_{01}$ = 2.52). There was also no significant effect of awareness of behavioural change on mean $D_{KL}$ (t (26.14) = 1.06, *p* = 0.298, $\xi$ = 0.18, ${BF}_{01}$ = 1.87). These results indicate that participants were influenced by the choices of the other to the same degree, regardless of whether they believed that the other participants were real, or whether they noticed their own behaviour changing throughout the task.

### Gender Differences in Individual Task Parameters

We also conducted exploratory analyses to determine whether there were any gender differences in task parameters. Independent-measures t-tests revealed no significant gender difference in log *k* in the Study 1 (t (44) = -1.70, *p* = 0.096, d = -0.50, ${BF}_{01}$ = 1.07), or Study 2 samples (t (96) = 1.48, *p* = 0.142, d = 0.31, ${BF}_{01}$ = 1.76). There was also no significant gender difference in log $\beta$ for the Study 1 sample (t (44) = -0.56, *p* = 0.580, d = -0.17, ${BF}_{01}$ = 3.01), although this difference was significant in the Study 2 sample (t (96) = 2.76, *p* = 0.007, d = 0.57, ${BF}_{10}$ = 5.89), whereby females (mean = -0.26, SD $\pm$ 0.37) made noisier choices than males (mean = -0.07, SD $\pm$ 0.37).

### Relationship Between AQ Subscales and Contagion

To determine whether there were any significant relationships between $D_{KL}$, and the subscales of the AQ, data were collapsed across the two NT samples and entered into regression analyses (see Table 1 for descriptive statistics for collapsed AQ data). Two regression analyses are conducted here, separately for more impulsive and more patient other agents. $D_{KL}$ is entered as the outcome variable, and the predictors are a binary group variable (Study 1/Study 2), accuracy, and the subscales of the AQ (Table 3).

**Table 3:** Statistics for the individual predictors (group, accuracy, and AQ subscales) entered into the regression model to predict $D_{KL}$

	Impulsive			Patient		
	$\beta$	T	*P*	$\beta$	t	*p*
Study group	1.36	.85	.395	2.06	1.31	.193
Accuracy	.12	1.63	.105	.15	1.94	.055
AQ—Social Skills	.02	.29	.776	− .07	− .91	.362
AQ—Attention Switching	− .01	− .07	.942	− .04	− .50	.616
AQ—Attention to Detail	.02	.28	.777	− .09	− 1.52	.131
AQ—Communication	− .00	− .07	.948	.13	1.78	.075
AQ—Imagination	− .04	− .58	.561	.08	1.22	.226

For the more impulsive agent, the overall model explained 0.8% of the variance in $D_{KL}$, and was not a significant predictor overall (F (7, 131) = 0.84, *p* = 0.556, ${BF}_{01}$ = 184.26). For the more patient agent, the overall model explained 6.9% of the variance in $D_{KL}$, and was a significant predictor overall (F (7, 131) = 2.46, *p* = 0.021, ${BF}_{10}$ = 0.40). In both models, none of the individual predictors significantly predicted $D_{KL}$, suggesting no relationship between AQ scores and contagion effects. This indicates that the level of autistic traits in our NT samples was not associated with contagion of TD value preferences.

#### Study 3

To allow for direct comparison between ASC and NT groups, a subset of NT participants (N = 12, 8 female, Study 2 sample) most closely matching the ages and genders of the Study 3 sample (N = 12, 8 female) were selected. The samples were also matched on the direction of the other (i.e., if the Study 3 participant had two negative others, participants were selected which had either two negative others, or a positive and negative other, but not two positive others). If multiple participants from the NT sample matched a participant in the ASC sample, a participant ID was selected at random.

The two groups were compared using Yuen’s robust independent-samples t-tests. The ASC group scored significantly higher on the majority of the AQ subscales, but there were no significant group differences in age, log *k*, log β, $D_{KL}$, or accuracy. See Table 4 for descriptive statistics and t-test statistics for these analyses.

**Table 4:** Descriptive statistics (mean (SD)), and comparisons (Yuen’s robust independent-measures t-tests) between the ASC and matched NT sample for: age, log *k*, log β, $D_{KL}$, accuracy and AQ subscales

	ASC	NT	t	df	*p*	$\xi$	*BF*
Age	22.33 (3.63)	22.42 (3.55)	.07	14.00	.948	.02	2.68
Log *k*	− 2.28 (.78)	− 1.57 (.81)	1.69	12.2	.116	.50	.53
Log β	− .08 (.52)	− .32 (.22)	1.39	10.74	.193	.51	1.24
$D_{KL}$– impulsive (log *k* + 1)	1.50 (3.31)	.41 (.85)	.51	6.21	.627	.23	1.68
$D_{KL}$- patient (log *k* − 1)	1.36 (3.57)	.45 (1.57)	.01	9.50	.992	.00	2.12
Accuracy-impulsive (log *k* + 1)	75.33 (8.92)	75.28 (11.50)	.37	10.85	.722	.12	2.59
Accuracy-patient (log *k* − 1)	79.72 (12.75)	77.78 (8.80)	.90	13.77	.383	.22	2.50
AQ Social Skills	29.83 (4.13)	19.92 (3.70)	6.40	13.74	< .001*	.87	4096.01
AQ Attention Switching	33.17 (3.66)	23.58 (3.20)	6.99	13.87	< .001*	.97	14,615.95
AQ Attention to Detail	29.42 (4.58)	24.92 (4.83)	2.10	13.98	.054	.57	.41
AQ Communication	31.67 (3.89)	20.00 (4.31)	6.42	14.00	< .001*	.91	19,376.95
AQ Imagination	25.42 (3.83)	19.83 (3.71)	3.06	13.15	.009*	.80	22.21
AQ Total	149.50 (14.39)	108.25 (11.96)	7.46	13.84	< .001*	.88	72,616.40

BF is ${BF}_{10}$ for significant analyses and ${BF}_{01}$ for non-significant analyses

* is for significant analyses

As the group differences in $D_{KL}$ and accuracy were not significant, equivalence Bayesian independent-measures t-tests were also run on the data to determine whether the samples were significantly equivalent on these variables. Equivalence bounds were set between -0.7 and 0.7 (determined by a power analysis for 33% power with a sample size of 12 in each group; Lakens, 2017).

**Fig. 5:**
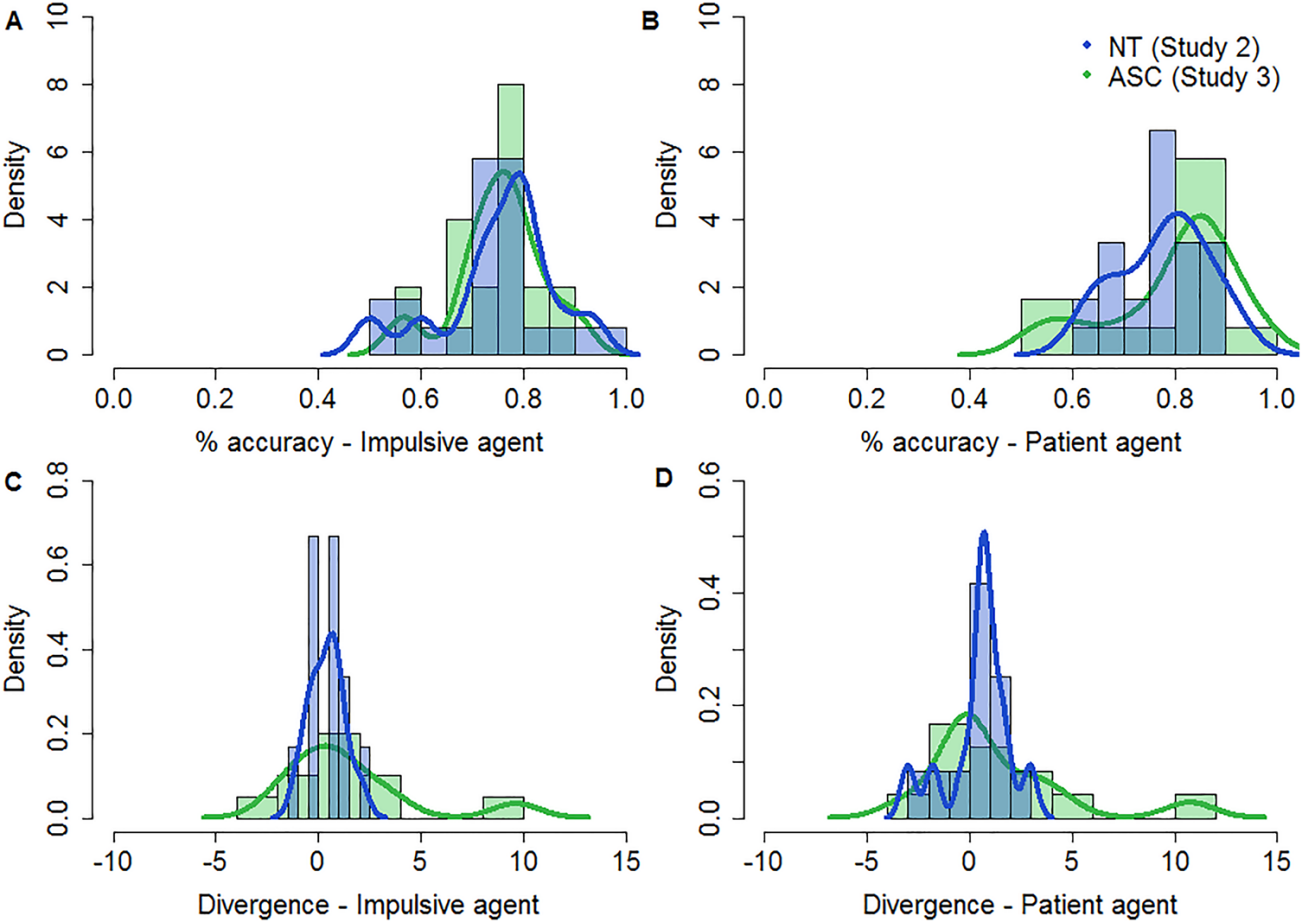
Histograms and density plots with comparisons between the NT subset and ASC sample. **a** Shows the comparison between samples for percentage accuracy for the impulsive agent, **b** shows the comparison between samples for percentage accuracy for the patient agent, **c** shows the comparison between samples for $D_{KL}$ for the impulsive agent, and **d** shows the comparison between samples for $D_{KL}$ for the patient agent

Bayesian support for equivalence was strong for accuracy for both impulsive (${BF}_{\in \notin }$ = 18.66), and patient (${BF}_{\in \notin }$ = 16.00) agents. For $D_{KL}$ Bayesian support for equivalence was moderate for impulsive (${BF}_{\in \notin }$ = 4.96), and patient (${BF}_{\in \notin }$ = 9.13) agents. See Fig. 5 for a comparison of the two samples. Together, these findings indicate that both contagion ($D_{KL}$) and accuracy for learning the preferences of another agent were statistically similar across the ASC and NT samples. This demonstrates that despite clear differences in social skills, autistic participants were able to learn the preferences of others and were influenced by them in the same way as NT controls.

## Discussion

Across three studies, we examined contagion of value preferences on a TD task, and whether contagion correlates with autistic traits and is disrupted in autistic adults. In both of our NT samples, we found significant contagion effects (i.e., a change in value preference after exposure to another person’s distinct value preference). The strength of contagion was not associated with belief in the agent, accuracy of learning about the other agent, or autistic traits. Analyses also provided support for equivalent contagion and accuracy across the ASC sample and a matched subset of NT participants. These findings suggest that the ability to learn the value preferences of others is not different in autistic individuals and shows no relationship with autism traits in the NT population. These results add to the growing literature exploring social influence in ASC and questioning the extent of social deficits in ASC.

### Individual Differences in Contagion in NT Samples

In line with previous research, we found that learning the different discounting preferences of another agent led to significant shifts in the participant’s own discount rate in two independent NT samples (Garvert et al., 2015; Moutoussis et al., 2016; Nicolle et al., 2012). Across our two independent NT samples, contagion effects were clearly present when participants learned the preferences of more patient agents, with weaker evidence for contagion effects for more impulsive agents. This bias towards becoming more patient has also been demonstrated by Moutoussis et al. (2016) using a more advanced computational modelling approach and a similar TD contagion paradigm. However, it should be noted that bias towards becoming more patient replicates even when using a less advanced computational modelling approach that does not take into account the precision of the prior beliefs (see ‘normalised shift in discount rate’ in Supplemental Analyses). Effect sizes and Bayes Factors indicated that contagion is a strong and reproducible phenomenon; however, we were unable to associate individual differences in belief that the agent was real with the size of the contagion effect. There was anecdotal support for a relationship between how accurately someone can predict the choices of another agent and contagion in one of our NT samples. These findings highlight the need for further research investigating variability in the strength of contagion and why some individuals are more influenced by others.

### Contagion Effects in ASC

Contrary to predictions, subscale scores on the AQ were not significantly related to contagion in the two NT samples. Whilst we did find group differences in AQ scores (with higher scores for the Study 1 sample), we collapsed across samples for our regression analysis predicting contagion. Furthermore, using equivalence tests, we found equivalent contagion in our ASC sample (Study 3) and a subset of NT participants (Study 2 sample). These findings challenge the extent to which social influence differs between autistic and NT populations.

Previous research into social influence in ASC has focused on conformity (an explicit social influence effect; Asch, 1956), and the findings have been varied (Lazzaro et al., 2018; Van Hoorn et al., 2017; Yafai et al., 2014). Lower rates of conformity have been found in autistic children compared to typically developing controls for a line judgement task administered before and after participants received incorrect social information from the experimenter (i.e., *“Most people think”*; Yafai et al., 2014). In a public goods game with peers, decreased conformity has been found for antisocial peer influence in adolescents with high levels of autism traits (across NT and autistic samples), whereas comparable levels of prosocial peer influence were found across groups (Van Hoorn et al., 2017). Autistic adults (as in the present study) have also been shown to be as susceptible to conformity as NT controls in tests of word memory (Lazzaro et al., 2018). Here, we use a different form of social influence (i.e., contagion) in combination with a distinct value-based decision-making paradigm. In line with Lazzaro et al. (2018), and Bowler and Worley (1994), we found no significant differences in contagion between autistic and NT samples. In addition, we also fail to find a relationship between any of the AQ subscales and the strength of contagion in the two NT samples. Finally, our equivalence tests provide significant support for similarity between the ASC sample and a subset of NT participants, thus not relying on the absence of an effect to draw our conclusions. Together, these findings support the notion that social influence (both conformity and contagion) is unrelated to ASC.

### Learning About Others in ASC

Our results also suggest that autistic individuals learnt the value preferences of other agents just as accurately as NT participants. Difficulties learning the distinct perspectives of another agent, often referred to as Theory of Mind (ToM), has characterised social impairments in ASC (Baron-Cohen et al., 1985; Premack & Woodruff, 1978). Whilst multiple previous studies have suggested that autistic individuals struggle to correctly attribute mental states to others (Baron-Cohen et al., 1985), an increasing number of studies have shown that the performance of autistic individuals depends on multiple factors, including: comorbidities, task framing, and motivation (Frith & Happé, 1994; Hamilton, 2009; Keysar et al., 2003; Oakley et al., 2016; Peterson et al., 2013; Senju et al., 2009; Shamay-Tsoory et al., 2019). Our results provide further evidence that autistic individuals are able to learn the distinct preferences of another person. In particular, they suggest that at least when feedback is explicit, social impairments are not observed. It could be that without explicit feedback, autistic individuals would show impairment on the task. Indeed, research has found that social information is less salient for autistic individuals than NT individuals (Freeth et al. 2011), and predictive coding models of ASC have suggested that autistic individuals generate less precise internal prior beliefs about the world and rely more heavily on external sources of information (Karvelis et al., 2018; Lawson et al., 2017; Pellicano & Burr, 2012; Van De Cruys et al., 2014). The implicit nature of social cues makes it more difficult to generate robust internal priors about others’ beliefs, as these external sources of information are inherently noisy and less precise. Our results suggest that by using more explicit social feedback, deficits in learning about others were extinguished such that accuracy was comparable between ASC and NT samples. Future research could manipulate the consistency of the other agents to account for the greater noise seen in social behaviour and assess learning for ASC and NT samples.

### Caveats and Conclusions

Taken together, these findings demonstrate that learning the value preferences of other agents robustly shifts the value preferences of neurotypical participants (i.e., contagion). In contrast to our hypothesis, there was no reliable evidence of a relationship between autistic traits and contagion in NT samples, and both contagion and social learning was similar across ASC and NT samples.

However, it is important to highlight two potential limitations. First, studies exploring a range of behavioural measures have shown that it is difficult to link individual differences in questionnaires with task data due to differences in variability (Frey et al., 2017; Hedge et al., 2017; Palminteri & Chevallier, 2018; Pedroni et al., 2017). Behavioural tasks (such as discounting tasks) produce replicable results because between-subject variability is low, which results in low reliability for measuring individual differences (Hedge et al., 2017). Nevertheless, similar research by Molleman et al. (2019) and Reiter et al. (2019) has found that the strength of contagion correlated with individual differences in social conformity and social integration values respectively. Whilst variability in our collapsed sample was similar to that of previous research (e.g., Goris et al., 2019, who reported an SD of 14.47, compared to 15.34 in our collapsed sample), we did not compare differences in contagion between high and low AQ scorers in our study. Future research could seek to recruit participants both with high and low AQ scores, in order to determine whether group differences exist here. Much of the data in our two NT samples was also collected from a university population, and further research should explore whether relationships between contagion and individual differences in AQ scores become clearer if all participants are recruited from the general population.

Second, it is possible that the lack of a significant group difference (i.e., ASC versus NT subset), and the significant support for similarity between groups reflects under sampling of the autistic population (N = 12). However, power calculations indicated that only four participants would be required to demonstrate a contagion effect (with 80% power) based on previous studies, and thus our study was well-powered. Nevertheless, future studies could seek to replicate the effects in a larger sample of ASC participants, given the very large effect sizes shown in previous contagion studies and the significant similarity between ASC and NT samples. We believe an effect of contagion of value preferences in ASC suggest a social context where Bayesian beliefs are accurately formed, and our findings suggest that exploring social influence using a Bayesian approach is a promising avenue for future research into ASC.

A Medical Research Council Fellowship (MR/P014097/1), a Christ Church Junior Research Fellowship, and a Christ Church Research Centre Grant to PL supported this work, and the Max Planck Society supports the research of MG. We would like to thank Alkistis Karagounis, Mehreen Mirza, Shai Nash, Sophie Taylor, Josie Thompson, Ashley Allemeier, Talika Gamaarachchi Pathiranage Dona, Daniel Drew and Ayat Abdurahman for assistance with data collection. Also thank you to all the neurotypical and autistic participants for giving up their time.

## Supplementary Information

Below is the link to the electronic supplementary material.

Supplementary file1 (DOCX 93 KB)
