# Supplementary material for: Contagion of Temporal Discounting Value Preferences in Neurotypical and Autistic Adults
Source: J Autism Dev Disord. 2021 Apr 2;52(2):700–13. doi: 10.1007/s10803-021-04962-5 (PMC8813822; doi:10.1007/s10803-021-04962-5)
Supplement: Supplementary file 1 — Supplementary file1 (DOCX 93 KB) [file 10803_2021_4962_MOESM1_ESM.docx]

**Supplemental Information**

**Contagion of Temporal Discounting Value Preferences in Neurotypical and Autistic Adults**

**Louisa Thomas, Patricia L. Lockwood, Mona Garvert, & Joshua H. Balsters**

**Supplemental methods**

**Generation of choice pairs**

***Study 1***

To ensure accurate estimations of participants’ discount rates, choice pairs for all Self-trials were generated according to two methods: generative and adaptive. These were alternated across trials (as per Garvert et al., 2015). The generative method involved generating all possible pairs of amounts and delays. For each Self-block, 25 trials were selected that best matched the indifference points of 25 hypothetical participants with log *k* values evenly distributed between -4 and 0 (Garvert et al., 2015; Nicolle et al., 2012). All 50 Other-trial choice pairs were generated according to the same method, so that the options presented to participants best matched the indifference points of 50 hypothetical subjects with evenly distributed log *k* values.

The remaining 25 trials for each Self-block were generated according to an adaptive method, which relied on a Bayesian framework to produce precise estimations of discounting parameters. This method has been shown to produce more reliable estimations of *k* value with fewer trials required (Vincent & Rainforth, 2017). The participant’s initial prior belief about log *k* (at the start of the first block) was set to be normally distributed, with a mean of -2 and a standard deviation of 1. After each decision, Bayes rule was used to form the posterior by updating this prior. Choice pairs were generated which probed participant’s indifference points, in order to refine the prior.

***Study 2, and 3***

As in Study 1, choice pairs for all Self-trials were generated according to the same adaptive and generative methods, alternated across trials. For each Self-block, the generative method was used to generate all possible pairs of amounts and delays, and 15 trials were selected that best matched the indifference points of 15 hypothetical participants with log *k* values evenly distributed between -4 and 0. All Other-trial choice pairs (30 per block) were generated according to the same method (i.e., for each block, choices best matched the indifference points of 30 hypothetical subjects with evenly distributed log *k* values). The remaining 15 trials in each Self-block were generated according to the adaptive method, in the same format as Study 1.

**Supplemental analyses**

**Distribution of contagion variables**

In the Study 1 sample, $D_{KL}$ values for impulsive others were normally distributed (W = .95, *p* = .079), and $D_{KL}$ values for patient others were not normally distributed (W = .94, *p* = .013). In the Study 2 sample, $D_{KL}$ values for impulsive others were not normally distributed (W = .96, *p* = .009), and $D_{KL}$ values were normally distributed (W = .98, *p* = .193).

**Shift calculation**

Previous research has included analyses using shift variables (Garvert et al., 2015), and this is also calculated here for comparison. Normalised shift was calculated for both NT samples (Study 1 and Study 2) included in this paper (i.e. shifts in the same direction as the other agent are positive, and shifts in the opposite direction to the other agent are negative). Basic (Self after Other - Self before Other) and distance-controlled (as per Garvert et al., 2015) shift variables are included here. Distance controlled shift was calculated using the following equations:

1. Shift1 = $\frac{{Self2}_{logk}- {Self1}_{logk}}{{Other1}_{logk}- {Self1}_{logk}}$
2. Shift2 = $\frac{{Self3}_{logk}- {Self2}_{logk}}{{Other2}_{logk}- {Self2}_{logk}}$

The log *k* value from the last trial of each block was used here, as discounting parameters are stable by this point. The log *k* values calculated for the Other blocks, were calculated from participants’ choices on behalf of the other agent. Supplemental Equation (1) shows the calculation of shift at Self2 away from Self1 and towards Other1. Supplemental Equation (2) shows this shift at Self3, away from Self2 and towards Other2. Shift variables were sorted into shift for impatient (i.e., more positive) and patient (i.e., more negative) other agents (based on the direction of the model, i.e., ±1 of the participant’s log *k* value).

To determine whether basic and distance-controlled normalised and absolute shifts in log *k* were significantly greater than zero, separate one-sample t-tests were run for more impulsive (log *k* +1) and more patient (log *k* -1) other agents. If data were not normally distributed (determined by Shapiro-Wilk tests of normality), Wilcoxon signed-rank tests were used.

**Normalised shift in discount rate**

In both NT samples, basic and distance-controlled normalised shift was significantly greater than zero for patient agents only. See Supplemental Figure 1 for descriptive statistics for all shift variables.

***Study 1***

Basic normalised shift in log *k* towards the more impulsive other was normally distributed (W = .96, *p* = .141), and was not significantly greater than zero (t (43) = 1.93, *p* = .060, $\mathcal{d}$ = .29, ${BF}_{01}$ = 1.12). Basic shift towards the more patient other was not normally distributed (W = .93, *p* = .010) and was significant (W (47) = 1010.00, *p* < .001, $\mathcal{d}$ = .68, ${BF}_{10}$ = 1007.63). Distance-controlled shift was not normally distributed (W = .72, *p* < .001) and was not significantly greater than zero (W (43) = 638.00, *p* = .097, $\mathcal{d}$ = .18, ${BF}_{01}$ = 3.15) for the impulsive agent. For the patient agent, distance-controlled shift was also not normally distributed (W = .29, *p* < .001), but was significantly greater than zero (W (47) = 930.00, *p* < .001, $\mathcal{d}$ = -.09, ${BF}_{10}$ = .19).

***Study 2***

In the Study 2 sample, basic shift was not normally distributed (W = .94, *p* < .001), and was not significantly greater than zero for the impulsive agent (W (97) = 2683.00, *p* = .362, $\mathcal{d}$ = .04, ${BF}_{01}$ = 8.18). Basic shift for the more patient agent was normally distributed (W = .98, *p* = .294), and was significantly greater than zero (t (94) = 4.31, *p* < .001, $\mathcal{d}$ = .44, ${BF}_{10}$ = 438.35). Distance-controlled shift was not normally distributed for the more impulsive agent in this sample (W = .48, *p* < .001), and was also not significantly greater than zero (W (97) = 2670.00, *p* = .387, $\mathcal{d}$ = .02, ${BF}_{01}$ = 8.70). Distance-controlled shift was also not normally distributed for the more patient other agent in this sample (W = .81, *p* < .001), but was significantly greater than zero (W (94) = 3308.00, *p* < .001, $\mathcal{d}$ = .31, ${BF}_{10}$ = 8.15).

**Contagion when accounting for uncertainty (**$\boldsymbol{D}_{\boldsymbol{KL}}$**) versus shift**

Bayes factors were compared for normalised distance-controlled shift (as per Garvert et al., 2015), which controls for the distance between Self and other in the calculation, and $D_{KL}$, which controls for the participants’ certainty in their belief in both blocks. This was to determine which variable resulted in the largest effect (Bayes factors in support of an effect (${BF}_{10}$) were used here for comparison purposes). In both NT samples, an effect of contagion was more likely when using the normalised $D_{KL}$ measure versus distance-controlled normalised shift.

***Study 1***

For the impulsive agent in this sample, an effect of contagion was 3.44 times more likely when using $D_{KL}$ to measure contagion (${BF}_{10}$ = 1.10) than when using the shift variable (${BF}_{10}$ = .32). Contagion was also greater (x 1499.90) when using $D_{KL}$ (${BF}_{10}$ = 284.98) versus shift (${BF}_{10}$ = .19) for the patient agent in this sample.

***Study 2***

In this sample, an effect of contagion was twice as likely when using the $D_{KL}$ variable (${BF}_{10}$ = .24) to measure contagion, versus shift (${BF}_{10}$ = .12) for the impulsive agent, and 5.12 times more likely when using $D_{KL}$ (${BF}_{10}$ = 41.76) versus shift (${BF}_{10}$ = 8.15) for the patient agent.


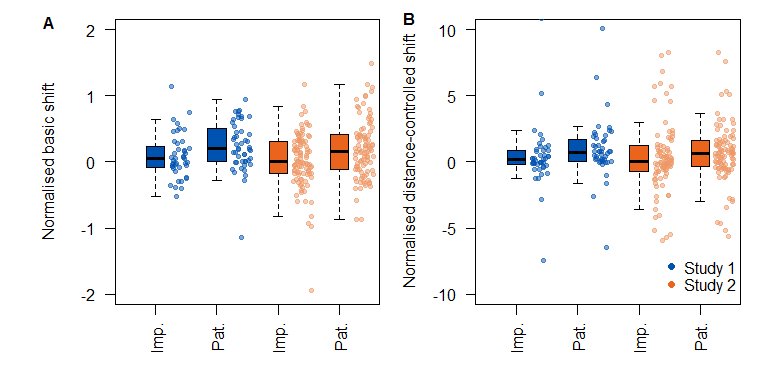


**Supplemental Fig. 1** Descriptive statistics for all shift variables for the two neurotypical samples (Study 1 and Study 2) are presented here, split for more impulsive (i.e., Imp., positive, log *k* +1) and more negative (i.e., Pat., negative, log *k* -1) agents. A positive value indicates a shift in value preference towards the other agent (for example, becoming more impulsive after being exposed to an impulsive other) and a negative value indicates a shift in behaviour away from the other agent (for example, becoming more patient after learning the preferences of a more impulsive other). (A) shows normalised basic shift variables, and (B) shows normalised distance-controlled variables.

**Supplemental Table 1.** Descriptive statistics for AQ subscale scores and total scores for both NT samples (Study 1 and Study 2), and the ASC sample (Study 3) using binary scoring (Baron-Cohen et al., 2001), following exclusions.

|  | Study 1 | | | Study 2 | | | Study 3 | | |
| --- | --- | --- | --- | --- | --- | --- | --- | --- | --- |
|  | Mean | SD | Range | Mean | SD | Range | Mean | SD | Range |
| AQ Social Skills | 5.20 | 1.76 | 1-9 | 2.54 | 1.97 | 0-8 | 6.92 | 1.62 | 4-9 |
| AQ Attention Switching | 5.09 | 1.84 | 2-9 | 4.67 | 2.08 | 1-10 | 8.75 | 1.14 | 7-10 |
| AQ Attention to Detail | 4.83 | 1.60 | 2-9 | 5.21 | 2.23 | 0-10 | 7.17 | 1.99 | 4-10 |
| AQ Communication | 4.74 | 1.45 | 2-7 | 2.23 | 1.94 | 0-8 | 8.08 | 1.44 | 5-10 |
| AQ Imagination | 5.07 | 1.70 | 1-8 | 2.32 | 1.82 | 0-9 | 5.50 | 1.68 | 3-9 |
| AQ TOTAL | 24.91 | 3.85 | 19-36 | 16.97 | 6.19 | 5-36 | 36.42 | 5.05 | 27-43 |

**Supplemental Table 2.** Presented here are the Bayes factors and interpretations for all analyses with the Study 1 (NT), Study 2 (NT), and Study 3 (ASC) samples and the comparison samples (NT subsample and ASC sample) in the order presented in the paper (main paper, followed by Supplemental Results) to allow for direct comparison.

|  | Study 1 (NT) | Study 2 (NT) | Studies 1 and 2 combined | Comparison (NT subset and ASC) |
| --- | --- | --- | --- | --- |
| One-sample t-test/Wilcoxon singed-rank test - $\boldsymbol{D}_{\boldsymbol{KL}}$, impulsive (log *k* +1) | $\boldsymbol{BF}_{\boldsymbol{10}}$ **= 1.10**  **Anecdotal** | ${BF}_{01}$ = 4.10  Moderate | - | - |
| One-sample t-test/Wilcoxon singed-rank test - $\boldsymbol{D}_{\boldsymbol{KL}}$, patient (log *k* -1) | $\boldsymbol{BF}_{\boldsymbol{10}}$ **= 284.98**  **Extreme** | $\boldsymbol{BF}_{\boldsymbol{10}}$ **= 41.76**  **Strong** | - | - |
| Robust repeated-measures t-test – Effect of direction (+/- log *k*) on $\boldsymbol{D}_{\boldsymbol{KL}}$ | $\boldsymbol{BF}_{\boldsymbol{10}}$ **= .91**  **Anecdotal support for H0** | ${BF}_{01}$ = 2.95  Anecdotal | - | - |
| Repeated-measures t-test – Effect of direction (+/- log *k*) on percentage accuracy | $\boldsymbol{BF}_{\boldsymbol{10}}$ **= 2.60**  **Anecdotal** | $\boldsymbol{BF}_{\boldsymbol{10}}$ **= 3.51**  **Moderate** | - | - |
| Correlation - $\boldsymbol{D}_{\boldsymbol{KL}}$ and accuracy, impulsive (log *k* +1) | ${BF}_{01}$ = 4.97  Moderate | $\boldsymbol{BF}_{\boldsymbol{10}}$ **= 4.56**  **Moderate** | - | - |
| Correlation - $\boldsymbol{D}_{\boldsymbol{KL}}$ and accuracy, patient (log *k* -1) | ${BF}_{01}$ = 2.86  Anecdotal | ${BF}_{01}$ = 2.79  Anecdotal | - | - |
| Independent-measures t-test – Effect of belief in manipulation on mean $\boldsymbol{D}_{\boldsymbol{KL}}$ | - | ${BF}_{01}$ = 2.52  Anecdotal | - | - |
| Independent-measures t-test – Effect of awareness of manipulation on mean $\boldsymbol{D}_{\boldsymbol{KL}}$ | - | ${BF}_{01}$ = 1.87  Anecdotal | - | - |
| Independent-measures t-test – Gender difference in log *k* | ${BF}_{01}$ = 1.07  Anecdotal | ${BF}_{01}$ = 1.76  Anecdotal | - | - |
| Independent-measures t-test – Gender difference in log β | ${BF}_{01}$ = 3.01  Moderate | $\boldsymbol{BF}_{\boldsymbol{10}}$ **= 5.89**  **Moderate** | - | - |
| Regression – Group, accuracy, and AQ subscales predicting $\boldsymbol{D}_{\boldsymbol{KL}}$, impulsive (log *k* +1) | - | - | ${BF}_{01}$ = 184.26  Extreme | - |
| Regression – Group, accuracy, and AQ subscales predicting $\boldsymbol{D}_{\boldsymbol{KL}}$, patient (log *k* -1) | - | - | $\boldsymbol{BF}_{\boldsymbol{10}}$ **= .40**  **Anecdotal support for H0** | - |
| Yuen’s robust Independent-samples t-test – Group difference in log *k* | - | - | - | ${BF}_{01}$ = 2.68  Anecdotal |
| Yuen’s robust Independent-samples t-test – Group difference in log $\boldsymbol{\beta}$ | - | - | - | ${BF}_{01}$ = .53  Anecdotal support for H0 |
| Yuen’s robust Independent-samples t-test – Group difference in $\boldsymbol{D}_{\boldsymbol{KL}}$, impulsive (log *k* +1) | - | - | - | ${BF}_{01}$ = 1.24  Anecdotal |
| Yuen’s robust Independent-samples t-test – Group difference in $\boldsymbol{D}_{\boldsymbol{KL}}$, patient (log *k* -1) | - | - | - | ${BF}_{01}$ = 1.68  Anecdotal |
| Yuen’s robust Independent-samples t-test – Group difference in % accuracy, impulsive (log *k* +1) | - | - | - | ${BF}_{01}$ = 2.59  Anecdotal |
| Yuen’s robust Independent-samples t-test – Group difference in % accuracy, patient (log *k* -1) | - | - | - | ${BF}_{01}$ = 2.50  Anecdotal |
| Yuen’s robust Independent-samples t-test – Group difference in AQ Social Skills subscale | - | - | - | $\boldsymbol{BF}_{\boldsymbol{10}}$ **= 4096.01**  **Extreme** |
| Yuen’s robust Independent-samples t-test – Group difference in AQ Attention Switching subscale | - | - | - | $\boldsymbol{BF}_{\boldsymbol{10}}$ **= 14615.95**  **Extreme** |
| Yuen’s robust Independent-samples t-test – Group difference in AQ Attention to Detail subscale | - | - | - | ${BF}_{01}$ = .41  Anecdotal support for H1 |
| Yuen’s robust Independent-samples t-test – Group difference in AQ Communication subscale | - | - | - | $\boldsymbol{BF}_{\boldsymbol{10}}$ **= 19376.95**  **Extreme** |
| Yuen’s robust Independent-samples t-test – Group difference in AQ Imagination subscale | - | - | - | $\boldsymbol{BF}_{\boldsymbol{10}}$ **= 22.21**  **Strong** |
| Yuen’s robust Independent-samples t-test – Group difference in AQ total score | - | - | - | $\boldsymbol{BF}_{\boldsymbol{10}}$ **= 72616.40**  **Extreme** |
| One-sample t-test/Wilcoxon singed-rank test – Basic normalised shift, impulsive (log *k* +1) | ${BF}_{01}$ = 1.12  Anecdotal | ${BF}_{01}$ = 8.18  Moderate | - | - |
| One-sample t-test/Wilcoxon singed-rank test – Basic normalised shift, patient (log *k* -1) | $\boldsymbol{BF}_{\boldsymbol{10}}$ **= 1007.63**  **Extreme** | $\boldsymbol{BF}_{\boldsymbol{01}}$ **= 438.35**  **Extreme** | - | - |
| One-sample t-test/Wilcoxon singed-rank test – Distance-controlled normalised shift, impulsive (log *k* +1) | ${BF}_{01}$ = 3.15  Moderate | ${BF}_{01}$ = 8.70  Moderate | - | - |
| One-sample t-test/Wilcoxon singed-rank test – Distance-controlled normalised shift, patient (log *k* -1) | $\boldsymbol{BF}_{\boldsymbol{10}}$ **= .19**  **Anecdotal support for H0** | $\boldsymbol{BF}_{\boldsymbol{10}}$ **= 8.15**  **Moderate** | - | - |

Note: $\mathrm{BF}_{01}$ is used for non-significant (i.e. *p* > .05) and indicates support for the null hypothesis (H0). $\mathrm{BF}_{10}$ is used for significant analyses (p < .05) and indicates support for the alternative hypothesis (H1).

**Supplemental References**

Baron-Cohen, S., Wheelwright, S., Skinner, R., Martin, J., & Clubley, E. (2001). The Autism Spectrum Quotient (AQ): Evidence from Asperger syndrome/high-functioning autism, males and females, scientists and mathematicians. *Journal of Autism and Developmental Disorders, 31*(1), 5-17.

Garvert, M. M., Moutoussis, M., Kurth-Nelson, Z., Behrens, T. E. J. & Dolan, R. J. (2015). Learning-induced plasticity in medial prefrontal cortex predicts preference malleability. *Neuron, 85*(2), 418-428.
